# WNT signaling in human pluripotent stem cells promotes HDAC2-dependent epigenetic programs and development of retinoic acid-responsive mesoderm

**DOI:** 10.1016/j.stemcr.2026.102964

**Published:** 2026-06-18

**Authors:** Bao Q. Thai, Elizabeth J. Sargent, Stephanie A. Luff, Colin A. Fields, Jared M. Churko, Jonathan N. Young, Anthony Bosco, Christopher M. Sturgeon, Deepta Bhattacharya

**Affiliations:** 1 Department of Immunobiology, University of Arizona College of Medicine, Tucson, AZ 85724, USA; 2 Department of Stem Cell Biology and Regenerative Medicine, Icahn School of Medicine at Mount Sinai, New York, NY 10029, USA; 3 Department of Cellular and Molecular Medicine, University of Arizona College of Medicine, Tucson, AZ 85724, USA; 4 BIO5 Institute, University of Arizona, Tucson, AZ 85724, USA; 5 Department of Otolaryngology, University of Arizona, Tucson, AZ, USA; 6 Department of Surgery, University of Arizona College of Medicine, Tucson, AZ 85724, USA

**Keywords:** pluripotent stem cells, mesoderm, hematopoiesis, lymphopoiesis, WNT signaling, transcription, epigenetics

## Abstract

Human pluripotent stem cells (hPSCs) can be used as a scalable source of lymphocytes for adoptive cell therapies, contingent on the robust generation of definitive hematopoietic intermediates. Early WNT activation with CHIR99021 during mesoderm induction promoted the formation of KDR+ ALDH1A2+ mesoderm, which is essential for subsequent generation of T cells in a retinoic acid (RA)-dependent manner. Integrated scRNA-seq and ATAC-seq defined a WNT-dependent developmental trajectory from hPSCs to KDR+ ALDH1A2+ mesoderm. Gene regulatory network modeling predicted HDAC2 and E-box transcription factors as regulators of RA-responsive mesodermal differentiation downstream of WNT. HDAC2 knockout impaired, while HDAC2 overexpression enhanced KDR+ ALDH1A2+ mesoderm formation. E-box factor manipulation had no discernible effect. An orthogonal chemical screen confirmed that HDAC2 inhibition suppressed KDR+ ALDH1A2+ mesoderm, whereas modulating histone methylation enhanced their formation. These findings reveal mechanisms by which WNT signaling promotes RA-responsive mesoderm, and they suggest methods to improve the generation of lymphocytes from hPSCs.

## Highlights

• Early activation of WNT signaling in PSCs promotes formation of ALDH1A2+ mesoderm
• Formation of ALDH1A2+ mesoderm is associated with robust T cell production
• Single-cell profiling implicates HDAC2-dependent programs in formation of ALDH1A2+ mesoderm
• Genetic manipulation of HDAC2 alters formation of ALDH1A2+ mesoderm from PSCs

## Teaser

Thai and colleagues show that early activation of WNT signaling in PSCs promotes the formation of ALDH1A2+ mesoderm, which is associated with T cell potential. Single cell multi-omics profiling of differentiating cells identified candidate transcriptional and epigenetic effectors that promote this fate, including HDAC2. These data suggest strategies to enhance the generation of therapeutically useful hematopoietic cell types from PSCs.

## Introduction

Human pluripotent stem cells (hPSCs), including human embryonic stem cells (hESCs) and induced pluripotent stem cells (iPSCs), offer a scalable starting point for allogeneic adoptive lymphocyte therapies to treat cancer and autoimmune diseases (Cichocki et al., 2020; Nishimura et al., 2013; Themeli et al., 2013). hPSCs have unlimited self-renewal potential and, with the advent of targeted nuclease technology, are amenable to genetic engineering (Hockemeyer and Jaenisch, 2016). Genetic modifications in hPSCs, such as chimeric antigen receptor (CAR) integration for tumor targeting, can be stably maintained through differentiation (Themeli et al., 2013). Moreover, edits to ablate major immune recognition pathways may enable hPSC-derived cells to evade rejection and serve as a source for off-the-shelf allogeneic therapies (Gornalusse et al., 2017; Pizzato et al., 2024; Xu et al., 2019).

A critical bottleneck, however, remains the inefficient generation of hematopoietic progenitors with full lymphoid potential from hPSCs. Lessons from both primary human fetal hematopoiesis and hPSC modeling have demonstrated that multiple progenitors with lymphoid potential emerge during development, but many of these intermediates do not yield lymphocytes robustly (Luff et al., 2024). A critical population of KDR+ ALDH1A2+ mesoderm is associated with the emergence of retinoic acid (RA)-dependent definitive hematopoiesis and robust lymphopoiesis (Fernandez et al., 2026; Luff et al., 2022). These RA-responsive cells are critically dependent on WNT signaling during mesoderm differentiation (Luff et al., 2022; Sturgeon et al., 2014).

In this study, we examined the signaling, transcriptional, and epigenetic pathways downstream of WNT that promote RA-responsive mesoderm differentiation from hPSCs. Using CHIR99021, we confirm that early WNT activation in embryoid bodies generates KDR+ ALDH1A2+ mesoderm that promotes the generation of T cell progenitors in an RA-dependent manner. Multi-omics single-cell RNA- and ATAC-sequencing, pseudotime trajectory analysis, gene regulatory network modeling, and genetic manipulations identified HDAC2 as a regulator of RA-responsive mesoderm emergence downstream of WNT. A targeted small-molecule screen of epigenetic inhibitors revealed that selective modulation of histone methylation enhances the numbers of RA-responsive cells. These findings help provide an epigenetic and transcriptional explanation for how to generate RA-responsive mesoderm, an intermediate essential for subsequent definitive hematopoiesis.

## Results

### Early WNT signaling induces the generation of RA-responsive mesodermal progenitors capable of definitive hematopoiesis in hPSCs

**Figure 1:**
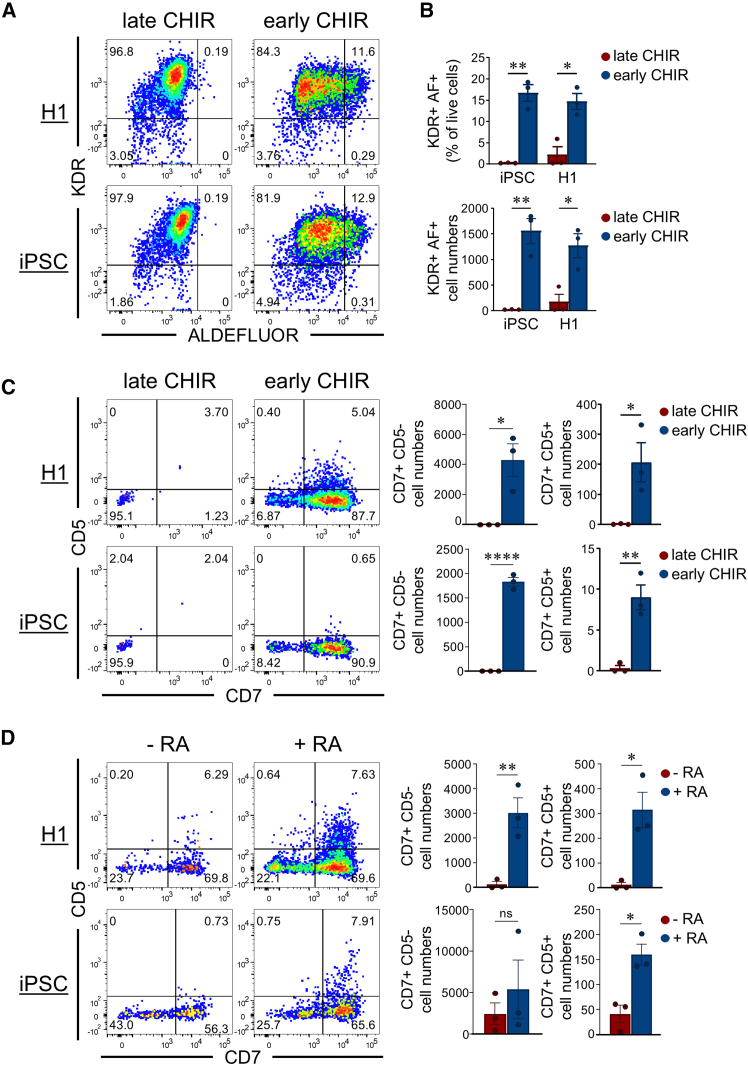
Early WNT signaling induces the generation of RA-responsive mesodermal progenitors capable of promoting definitive hematopoiesis in hPSCs (A) Representative flow cytometry plots showing expression of KDR and ALDEFLUOR (AF) in H1- and iPSC-derived mesodermal progenitors on day 3 of EB differentiation, with CHIR99021 added on day 0 (early CHIR) or on day 2 (late CHIR). (B) Quantification of the percentage of KDR+ AF+ cells (top) and total KDR+ AF+ cell numbers (bottom) in H1- and iPSC-derived cultures. Statistical analysis was performed using two-way ANOVA with Šídák multiple comparisons test, ^∗^*p* < 0.05, ^∗∗^*p* < 0.01, *n* = 3. Data are presented as mean ± SEM. Each circle represents an independent experiment. (C) Representative flow cytometry plots showing the expression of CD5 and CD7 in H1- and iPSC-derived T cell progenitors on day 14 of T cell differentiation from early versus late CHIR treated with RA on day 3 (left). Quantification of the total number of CD7^+^ CD5^−^ T/NK progenitors and CD7^+^ CD5^+^ T-committed progenitors in H1- (top right) and iPSC-derived (bottom right) cultures. Statistical analysis was performed using two-tailed *t* test, with ^∗^*p* < 0.05, ^∗∗^*p* < 0.01, ^∗∗∗∗^*p* < 0.0001, *n* = 3. Data are presented as mean ± SEM. Each circle represents an independent experiment. (D) Representative flow cytometry plots showing the expression of CD5 and CD7 in H1- and iPSC-derived T cell progenitors on day 14 of T cell differentiation from untreated and RA-treated progenitors (left). Quantification of the total number of CD7^+^ CD5^−^ T/NK progenitors and CD7^+^ CD5^+^ T-committed progenitors in H1- (top) and iPSC-derived culture (bottom). Statistical analysis was performed using two-tailed *t* test, with ^∗^*p* < 0.05, ^∗∗^*p* < 0.01, *n* = 3. Data are presented as mean ± SEM. Each circle represents an independent experiment.

In our previous studies, we identified a small but distinct ALDH1A2*+* population within early mesoderm that correlated with the emergence of RA-dependent definitive hematopoiesis, and found that this population was specified in a WNT-dependent manner (Luff et al., 2022). Titrating BMP4 and FGF concentrations between 10 and 50 ng/mL did not markedly impact the numbers of these cells. Recent protocols to generate multipotent hematopoietic stem and progenitor cells from hPSCs activate WNT signaling from the very beginning of mesoderm induction (Fowler et al., 2024; Ng et al., 2024). Therefore, to improve the efficiency of generating this population from hPSCs, we tested the impact of early WNT signal activation on the yields of ALDH1A2+ mesoderm. We treated hPSCs with CHIR99021, a glycogen synthase kinase 3 beta (GSK3β) inhibitor that activates canonical WNT signaling via stabilization of β-catenin, from day 0 (early CHIR) or day 2 (late CHIR) during a three-day embryoid body (EB) differentiation protocol (Figure S1A). On day 3, we assessed the emergence of RA-responsive mesoderm by flow cytometry using KDR as a mesodermal marker and ALDEFLUOR (AF) as a reporter of the RA-processing enzyme aldehyde dehydrogenase (ALDH) (gating strategy in Figure S1B). Flow cytometric quantification showed that CHIR99021 significantly increased the generation of KDR+ AF+ mesodermal progenitors on day 3 (Figures 1A and 1B), and CD34^+^ hematopoietic progenitors on day 16 of EB cultures (Figure S1C), derived from both H1 hESCs and an iPSC line derived from a human tonsillar B cell. Late CHIR conditions yielded similar frequencies and numbers of KDR+ AF+ mesoderm as when CHIR99021 was excluded entirely (Figure S1D). To confirm that these KDR+ AF+ and subsequent CD34^+^ progenitors were correctly specified for definitive hematopoiesis, we performed T cell assays (Figure S1A, gating strategy in Figure S1E). When RA was added at day 3, early CHIR cultures robustly yielded CD7^+^ NK/T progenitors and T-committed CD5^+^ CD7^+^ progenitors, while late CHIR cultures did not (Figure 1C). Excluding RA from early CHIR cultures markedly reduced T-committed progenitors from both H1 hESCs and iPSCs (Figure 1D). These results indicate that early WNT signaling promotes the development of RA-responsive mesoderm important for the subsequent formation of definitive hematopoietic progenitors.

### Early WNT signaling induces transcriptional changes associated with definitive hematopoiesis

**Figure 2:**
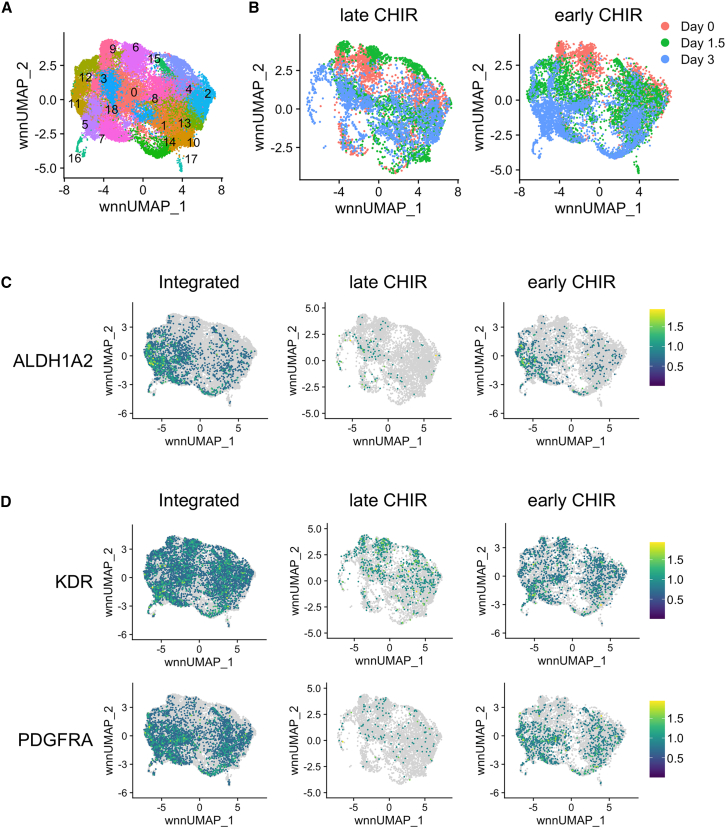
Early WNT signaling induces transcriptional changes associated with RA-responsive mesoderm formation (A) UMAP plot of combined single-cell ATAC- and RNA-seq data integrated using the weighted nearest neighbor (WNN) method, showing distinct clusters of cells during the first 3 days of EB differentiation with CHIR99021 added on day 0 (early CHIR) or day 2 (late CHIR). (B) UMAP plots showing the progression of differentiation over time for early CHIR and late CHIR conditions. (C) Relative expression of ALDH1A2 in an integrated UMAP or separated as early CHIR or late CHIR conditions. Color bar is based on scaled relative expression. Gray indicates no detectable expression. (D) Relative expression of mesoderm markers *KDR* and *PDGFRA* in an integrated UMAP or separated as early CHIR or late CHIR conditions.

To define how early WNT signaling promotes KDR+ ALDH1A2+ mesoderm formation, we performed combined single-cell RNA sequencing (scRNA-seq) and single-cell ATAC sequencing (scATAC-seq) analysis on H1 hESCs during each of the first three days of differentiation. Analysis of integrated scRNA- and scATAC-seq data identified 18 distinct clusters (Figure 2A). Compared to late CHIR cells, early CHIR cells diverged transcriptionally and epigenetically by day 1.5 (Figure 2B), and these differences were magnified by day 3 (Figure S2A). ALDH1A2 expression was seen primarily in KDR+ PDGFRA+ mesoderm progenitors in early CHIR cultures on day 3, corroborating our flow cytometric data (Figures 2C and 2D). Expression of ALDH1A1 and ALDH1A3 was minimal (Figure S2B*).* HOXA gene expression (HOXA9 and HOXA10) was observed in ALDH1A2+ cells on day 3 of early CHIR cultures, further supporting the role of WNT signaling in regulating the formation of definitive hematopoietic progenitors (Figure S2C) (Dou et al., 2016; Ng et al., 2016).

### *In silico* gene perturbations identified transcriptional and epigenetic regulators in the development of ALDH1A2+ KDR+ RA-responsive mesoderm

**Figure 3:**
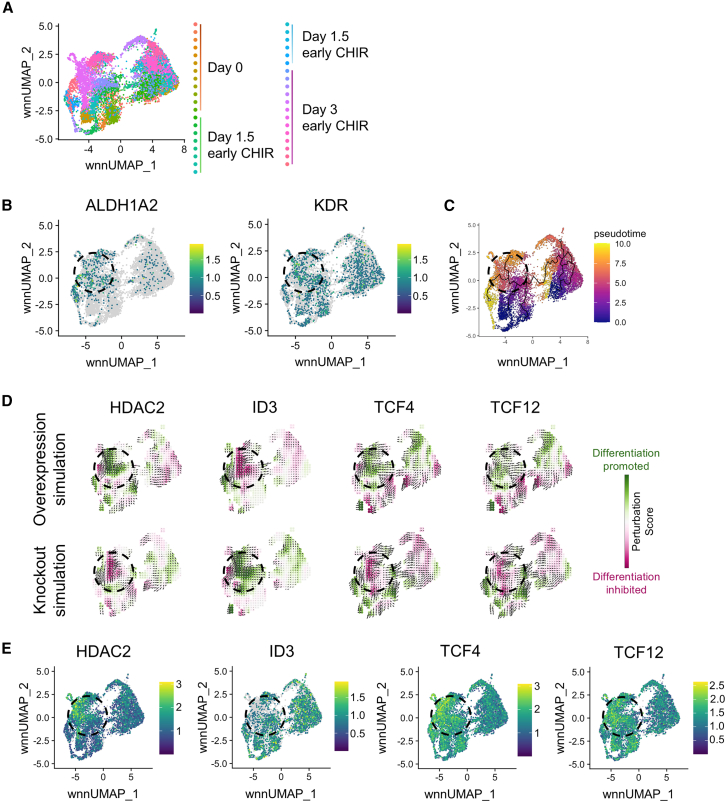
*In silico* gene perturbations identify transcriptional and epigenetic regulators in the development of ALDH1A2+ KDR+ RA-responsive mesodermal progenitors (A) UMAP plot showing integrated scATAC- and scRNA-seq data from day 0 (undifferentiated), day 1.5, and day 3 of EB differentiation treated with CHIR99021 on day 0 (early CHIR). (B) Feature plots of ALDH1A2 and KDR relative expression to localize RA-dependent mesodermal progenitors on the UMAP embedding. Dashed circles represent cells enriched in ALDH1A2 and KDR. (C) Pseudotime analysis using Monocle 3.0 visualized on the same UMAP. Black lines indicate inferred differentiation trajectories from undifferentiated pluripotent stem cells toward differentiated RA-responsive mesodermal progenitor populations. (D) CellOracle *in silico* simulation to predict the effects of overexpression and knockout of HDAC2, ID3, TCF4, and TCF12 on differentiation trajectories. Colors represent perturbation score, indicating predicted promotion (green, positive perturbation score) or inhibition (magenta, negative perturbation score) of differentiation upon simulated gene perturbation. (E) Relative expression of HDAC2, ID3, TCF4, and TCF12 in UMAP space.

To identify regulators of the development of WNT-induced ALDH1A2+ KDR+ mesoderm, we reconstructed our UMAPs to focus only on early CHIR cells (Figures 3A and 3B). Using Monocle 3.0 (Cao et al., 2019), pseudotime analysis was performed to map developmental trajectories from day 0 to day 3 (Figure 3C). We next used CellOracle, which uses both scATAC-seq and scRNA-seq to predict gene regulatory networks that may be driving developmental transitions (Kamimoto et al., 2023). As internal validation of the method, *in silico* modeling predicted that genetic ablation of SP5, a known WNT negative regulator, would promote differentiation with a positive perturbation score along the developmental trajectory of day 0, day 1.5, and day 3 cells (Figure S3A) (Huggins et al., 2017).

We next examined other regulators also identified in our CellOracle analysis. ID3, TCF4, and TCF12 were predicted to strongly influence the transcriptional programs of day 1.5 cells from day 0 cells, while HDAC2 was predicted to be important for day 3 cells to develop from day 1.5 cells (Figure S3B). ID3, TCF4 (E2-2), and TCF12 (HEB) function as part of the ID-E protein network, in which ID proteins sequester TCF factors (E proteins) to regulate proliferation and lineage commitment, particularly during T and B cell development (Lazorchak et al., 2006; O’Riordan and Grosschedl, 1999; Roels et al., 2022). HDAC2 removes acetyl groups from histones, driving chromatin compaction and transcriptional repression, and has been shown to facilitate the endothelial-to-hematopoietic transition after mesodermal and hemogenic endothelial specification (Thambyrajah et al., 2018). *In silico* simulations predicted that TCF4, TCF12, and HDAC2 overexpression (OE) and ID3 knockout (KO) would promote differentiation toward day 3 ALDH1A2+ KDR+ mesoderm (Figure 3D).

To consider the potential validity of these predictions, we examined the gene expression of these potential regulators throughout our scRNA-seq data. HDAC2 was highly expressed in the day 3 ALDH1A2+ and KDR+ clusters, while ID3 was mostly absent in these cells. TCF4 and TCF12 expressions remained largely unchanged during the differentiation process (Figure 3E). Together, these data predict a regulatory network, in which TCF4, TCF12, ID3, and HDAC2 act as potential early drivers in the development of WNT-induced ALDH1A2+ KDR+ mesoderm.

### Functional validation confirmed HDAC2 as a key regulator required for RA-responsive mesoderm differentiation

To validate the functional roles of potential regulators in RA-responsive mesodermal differentiation identified through our CellOracle analysis, we performed CRISPR-Cas9-mediated KOs of each gene and examined the impacts on differentiation in H1 hESCs. We electroporated H1 cells with Cas9 and guide RNAs (gRNAs) targeting HDAC2, ID3, TCF4, and TCF12, individually, or TCF4 and TCF12 in combination. We then subjected these edited cells to EB differentiation in the presence of CHIR99021 at day 0 to generate RA-responsive mesodermal progenitors. On day 3, differentiated EBs were dissociated into single cells and sorted by fluorescence-activated cell sorting (FACS) into KDR+ AF+ and KDR+ AF- mesodermal progenitor populations. Genomic DNA was extracted from sorted populations, followed by PCR amplification and Nanopore sequencing to assess frameshift mutation (FS) frequencies in each sorted population compared with day 0 undifferentiated cells.

**Figure 4:**
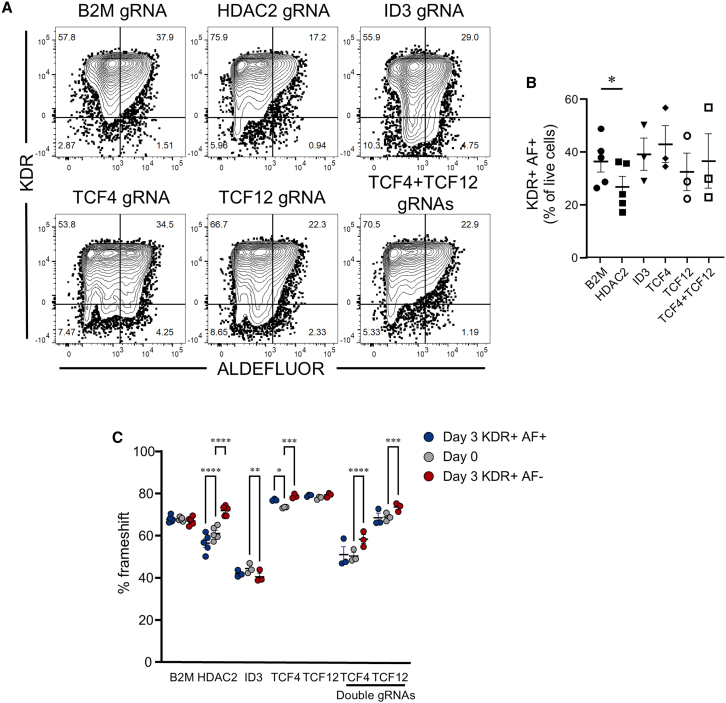
Functional validation of HDAC2 as a key regulator required for RA-responsive mesodermal progenitor differentiation (A) Representative flow cytometry plots showing the differentiation of KDR+ AF+ mesodermal progenitors on day 3 of differentiation. H1 hESCs were electroporated with Cas9-gRNA complexes targeting HDAC2, ID3, TCF4, and TCF12, individually, or TCF4 and TCF12 in combination prior to EB differentiation in the presence of early CHIR. After 3 days, differentiated cells were sorted into KDR+ AF+ and KDR+ AF- populations via fluorescence-activated cell sorting (FACS). Genomic DNA was extracted, PCR-amplified, and sequenced using Nanopore sequencing to evaluate frameshift frequency. (B) Quantification of KDR+ AF+ mesodermal progenitor percentage. Statistical analyses were performed using one-way ANOVA with Dunnet multiple comparisons test. ^∗^*p* < 0.05, *n* = 3, except for B2M and HDAC2 where *n* = 5. Data are presented as mean ± SEM. Each symbol represents the value from an independent experiment. (C) Quantification of CRISPR-Cas9 gene-editing frameshift frequencies. Statistical analyses were performed using paired two-way ANOVA with Šídák multiple comparisons test. ^∗^*p* < 0.05, ^∗∗^*p* < 0.01, ^∗∗∗^*p* < 0.001, ^∗∗∗∗^*p* < 0.0001, *n* = 3, except for B2M and HDAC2 where *n* = 5. Data are presented as mean ± SEM. Each circle within a column represents the value from an independent differentiation.

Flow cytometry analysis revealed that KO cultures of HDAC2 subtly but consistently showed decreased percentages of KDR+ AF+ mesodermal progenitors compared with the control (B2M KOs) (Figures 4A and 4B), corroborating our prediction that HDAC2 promotes differentiation of these progenitors. In contrast, KOs of ID3, TCF4, TCF12, or TCF4 and TCF12 did not lead to statistically significant differences in differentiation compared with control cells. As an additional way to quantify the impact of these KOs, we quantified frameshift mutation frequencies in sorted populations and compared with those in undifferentiated day 0 cells (Figure 4C). For HDAC2 KO cultures, day 3 KDR+ AF+ cells had significantly lower frameshift mutation frequencies compared with both day 3 KDR+ AF- and day 0 starting cells, consistent with selection against cells that lack HDAC2. No such statistically significant changes were observed in KO cultures of B2M, ID3, TCF4, TCF12, or TCF4 and TCF12. To prove that this approach accurately estimates loss of function and expression, we performed intracellular flow cytometric analysis of HDAC2 protein. We observed that approximately half of the cells in HDAC2 KO cultures lacked detectable protein (Figure S4A), aligning well with the estimated frameshift mutation frequency.

**Figure 5:**
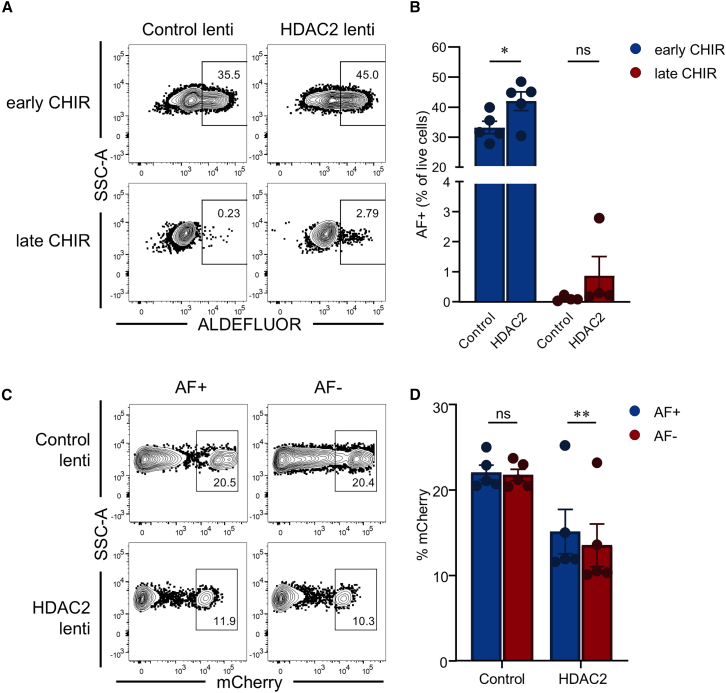
HDAC2 overexpression improves AF+ progenitor differentiation (A) Representative flow cytometry plots showing the differentiation of AF+ progenitors on day 3 of differentiation. H1 cells were transduced with either mCherry-expressing lentiviral vectors encoding HDAC2 or control (empty vector). Transduced cells were then subjected to the aforementioned differentiation protocol with CHIR99021 on day 0 (early CHIR) or on day 2 (late CHIR). (B) Quantification of AF+ progenitor percentage. Statistical analyses were performed using paired two-way ANOVA with Šídák multiple comparisons test. ^∗^*p* < 0.05, *n* = 5. Data are presented as mean ± SEM. Each circle within a column represents an independent differentiation. (C) Representative flow cytometry plots quantifying mCherry+ cells within AF+ and AF- progenitors. (D) Quantification of mCherry+ percentage. Statistical analyses were performed using paired two-way ANOVA with Šídák multiple comparisons test. ^∗∗^*p* < 0.01, *n* = 5. Data are presented as mean ± SEM. Each circle within a column represents an independent differentiation.

For further validation, we overexpressed HDAC2 in H1 hESCs using mCherry-containing lentiviral vectors and examined how differentiation is subsequently affected. Compared with controls, HDAC2 overexpression promoted AF+ cell differentiation in the presence of CHIR99021 (Figures 5A and 5B). Additionally, we quantified the percentages of mCherry+ cells in AF+ populations and compared with those in AF- cells. In HDAC2 overexpression cultures, AF+ cells had subtly but statistically significantly higher mCherry+ percentages compared with AF- cells, indicating selection for cells that have HDAC2 overexpressed (Figures 5C and 5D). The moderate increase in AF+ cell differentiation due to HDAC2 overexpression might be attributed to the modest 15% increase in HDAC2 protein levels from baseline (Figure S4B). Together, these results confirmed that HDAC2 marks and promotes differentiation of ALDH1A2+ RA-responsive mesoderm. We did not observe statistically significant effects of ID1 and ID3 transcription factor overexpression on AF+ progenitors (Figures S5A and S5B).

Because addition of CHIR99021 at late time points failed to yield AF + cells (Figure 1A), the data argue that WNT signaling likely acts upstream of HDAC2, which is expressed throughout the culture (Figure 3E). We, therefore, considered the possibility that WNT signaling establishes an early chromatin accessibility landscape that enables HDAC2 to drive transcription toward the AF+ fate. To test this possibility, we identified 4,034 genes associated with differentially accessible regions (DARs) in CHIR99021-treated cells at day 1.5 (Figure S6A). We then intersected this list with 2,206 differentially expressed genes (DEGs) between early- and late-CHIR cultures predicted to lie downstream of HDAC2 at day 3 (Figure S6A). We identified 708 genes that overlapped between these lists (Figure S6A). Pathway enrichment analysis of these genes revealed WNT signaling, as expected, as well as processes involved in adherence and tight junctions (Figure S6B), supporting a model in which the AF+ fate is driven by early WNT signaling, priming chromatin accessibility programs that are subsequently reinforced by HDAC2-dependent transcriptional regulation.

### Drug screening revealed common histone modifications as epigenetic regulations in the development of RA-responsive mesoderm

**Figure 6:**
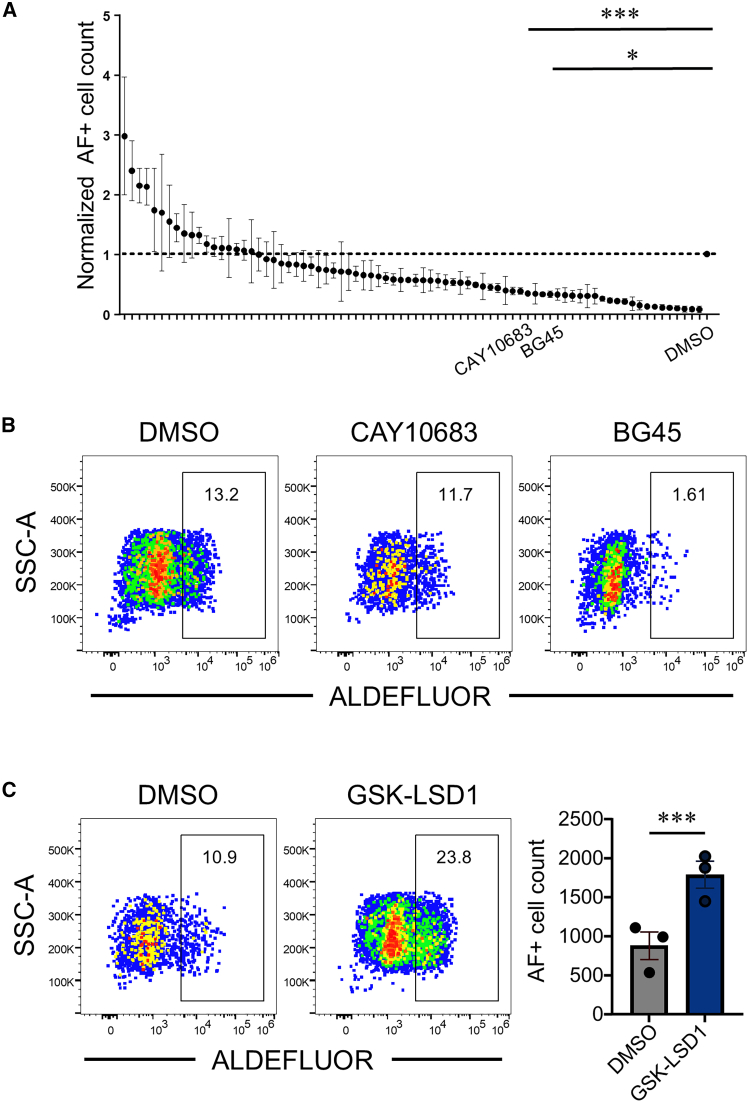
Drug screening reveals common histone modifications as epigenetic regulators in the development of RA-responsive mesodermal progenitors (A) Quantification of AF+ cells in day 3 EB differentiation cultures treated on day 0 of early CHIR differentiation with 10 *μ*M compounds from a library of 152 epigenetic small molecules. Values are normalized to the DMSO-treated controls. Statistical analyses were done using one-way ANOVA with Dunnet multiple comparisons test, ^∗^*p* < 0.05, ^∗∗∗^*p* < 0.001, *n* = 3. Data are presented as mean ± SEM from technical replicates. (B) Representative flow cytometry plots showing AF expression in day 3 cultures treated with DMSO (control) and two HDAC2 inhibitors, CAY10683 and BG45. (C) Representative flow cytometry plots showing AF expression after treatment with the top drug inhibitor GSK-LSD1 and DMSO (left). Quantification of absolute AF+ cell numbers across cultures treated with GSK-LSD1 and DMSO (right). Data are presented as mean ± SEM.

As an orthogonal method to identify epigenetic factors that regulate the differentiation of ALDH1A2+ mesoderm, we conducted a chemical screen using a 152-compound library of small-molecule epigenetic inhibitors. EBs were treated with individual compounds at 10 μM final concentrations on day 0 and subjected to CHIR99021-induced mesodermal induction. On day 3 of differentiation, AF+ cell numbers were quantified by flow cytometry and normalized to DMSO-treated controls. Most compounds had no significant impact on AF+ cell differentiation (Figure 6A; Table S2). However, CAY10683 and BG45, two known HDAC2 inhibitors, reduced the frequency of AF+ cells while maintaining their viability, corroborating the role of an HDAC2-dependent epigenetic program in promoting AF+ cells (Figures 6A, 6B, and S7A). On the other hand, the top drug that enhanced the numbers of AF+ cells was GSK-LSD1 (Figure 6C), an inhibitor of histone demethylation, highlighting an additional epigenetic mechanism that supports AF+ cell formation. Together, these data demonstrate that early WNT activation during mesoderm induction promotes RA-dependent progenitors in part through epigenetic programs, such as those downstream of HDAC2 and LSD1. To evaluate downstream impact, we examined T cell differentiation following treatment with Cay10683, BG45, and GSK-LSD1. Although these compounds significantly affected AF+ mesodermal progenitors at early stages, we observed that Cay10683 and BG45 did not affect, and GSK-LSD1 reduced CD34 output on day 16 of differentiation (Figure S7B). Additionally, when we seeded equivalent numbers of CD34^+^ cells derived from these differentiations, we did not observe significant differences in the production of committed CD7^+^ CD5^+^ T progenitors (Figures S7C and S7D). BG45 treatment resulted in a modest but significant reduction in CD7^+^ CD5^−^progenitors, while commitment to CD7^+^ CD5^+^ T progenitors remained unchanged.

## Discussion

The specification of definitive hematopoietic progenitors from hPSCs has remained a barrier for adoptive cell therapies. Recent studies have shown that WNT pathway activation is critical to guide cells down the correct initial developmental pathways that lead to RA-responsive mesoderm and subsequent definitive hematopoiesis (Luff et al., 2022; Ng et al., 2024; Sturgeon et al., 2014). Here, we investigated pathways downstream of WNT signaling that mark and promote the formation of RA-responsive, ALDH1A2-expressing mesodermal progenitors. Gene regulatory network analysis predicted that both HDAC2- and E-box transcription factors were likely involved in RA-responsive mesoderm specification. Yet functional validation experiments were only able to confirm the role of HDAC2. It is possible that the E-box protein-dependent programs may not be necessary for the formation of these mesodermal progenitors but may imprint subsequent lymphocyte potential. Studies in mice have shown that E-box transcription factors are critical for lymphopoiesis (Bain et al., 1994; 1997). Moreover, the reduction of E-box transcriptional programs may underlie age-dependent reductions in lymphopoiesis and function (Frasca et al., 2004a; 2004b).

We observed that HDAC2 promoted RA-responsive mesoderm only when WNT signaling was concurrently activated using CHIR99021. These data suggest that HDAC2 alone is insufficient to initiate RA-dependent mesodermal identity and instead functions in coordination with WNT-mediated transcriptional programs. One possible explanation is that HDAC2 facilitates the repression of lineage-inappropriate genes and perhaps also stabilizes chromatin accessibility at WNT target loci, thereby enhancing the transcriptional environment needed for ALDH1A2+ progenitor emergence. The impact of HDAC2 manipulation was relatively subtle, suggesting that partially redundant pathways such as other HDACs may compensate for RA-responsive mesoderm specification. These findings highlight the importance of both epigenetic state and signaling context in regulating definitive mesodermal fate. For future studies, combining targeted chromatin modulation with stage-specific signals may improve differentiation efficiency.

Finally, our small-molecule screen revealed that several distinct epigenetic modulators altered the generation of ALDH1A2+ mesodermal cells, such as GSK-LSD1, which inhibits LSD1 demethylase activity. Despite an enhancement of AF+ mesoderm, we noted a suppression of CD34^+^ progenitors emerging from these cultures. These data can potentially be explained by recent results showing that the ALDH1A2+ cells act non-autonomously to promote definitive hematopoiesis by other progenitors in the culture (Fernandez et al., 2026). Thus, enhancing ALDH1A2+ mesoderm may not proportionately increase definitive hematopoiesis if other progenitors are negatively impacted. HDAC2 or HDAC1 can form complexes with LSD1 and coREST to regulate distinct transcriptional programs (Aufhauser et al., 2021). When HDAC2 is deleted, HDAC1-LSD1-coREST complexes become more stable, with HDAC1 directly promoting LSD1 demethylase activity (Aufhauser et al., 2021; Nalawansha and Pflum, 2017). This suggests a model in which HDAC2 promotes RA-responsive mesoderm, potentially by inhibiting LSD1 demethylase activity via a decrease in HDAC1-LSD1-coREST complex formation. Future studies should include integrating these chromatin-targeting agents into directed differentiation protocols, with the aim of selectively activating and silencing gene programs that favor the formation of ALDH1A2+ mesoderm. Studies such as ours show how modern single cell multi-omics profiling can help guide these manipulations. These insights may accelerate the iterative optimization of protocols for definitive hematopoiesis and adoptive cell therapies.

## Resource availability

### Lead contact

Further information and requests for resources and reagents should be directed to Deepta Bhattacharya (deeptab@arizona.edu).

### Materials availability

Plasmids and cells generated in this study are available upon request and completion of a materials transfer agreement.

### Data and code availability

Single cell multi-omics data are available at the NCBI Sequence Read Archive, Bioproject number PRJNA1272807, according to FAIR principles.

## Acknowledgments

This work was supported by the 10.13039/100000002NIH grant nos. 5R01EB035491 (D.B. and C.M.S.), R01HL172940 (C.M.S.), T32AG058503 (B.Q.T.), R21AI176305 (A.B.), and P30CA023074 (for the Flow Cytometry Shared Resource). This work was also supported by the Gates Foundation (INV-1206188, INV-071091, and INV-002414), the Gootter-Jensen Foundation, and the BIO5 Institute. We thank the Flow Cytometry Core and Functional Genomics Core at the University of Arizona.

## Author contributions

Conceptualization, D.B. and B.Q.T.; funding acquisition, D.B., C.M.S., and B.Q.T.; methodology, B.Q.T., S.A.L., J.M.C., C.M.S., E.J.S., C.A.F., and A.B.; investigation, B.Q.T.; formal analysis, B.Q.T.; supervision, D.B. and C.M.S.; writing – original draft, B.Q.T.; writing – review and editing, D.B., S.A.L., C.M.S., and J.M.C.

## Declaration of interests

Sana Biotechnology has licensed intellectual property of D.B. and Washington University in St. Louis. Jasper Therapeutics and Inograft Therapeutics have licensed intellectual property of D.B. and Stanford University. D.B. served on an advisory panel for GlaxoSmithKline on COVID-19 therapeutic antibodies. D.B. served on the scientific advisory board for Hillevax. D.B. is a scientific cofounder of Aleutian Therapeutics. C.M.S. is an inventor in patent titled “Methods to obtain retinoic acid-dependent hematopoietic progenitors from human pluripotent stem cells” (International Publication No. WO2020154412 A1), pertaining to the application of the methodologies described in this manuscript. A.B. is the founder and equity holder of the startup company INSiGENe Pty Ltd. that is unrelated to this work. A.B. is a cofounder, equity holder, and director of the startup company Respiradigm Pty Ltd. that is unrelated to this work.

## STAR★Methods

### Key resources table

**Table:** 

REAGENT or RESOURCE	SOURCE	IDENTIFIER
**Antibodies**		

KDR–PE-Cy7	BioLegend	Cat# 359912; RRID: AB_2563552
CD13–PE-Dazzle™ 594	BioLegend	Cat# 301720; RRID: AB_2616764
CD14–PE-Cy7	BioLegend	Cat# 301814; RRID: AB_389353
CD45–FITC	BioLegend	Cat# 368508; RRID: AB_2566368
CD5–PE-Cy5	BioLegend	Cat# 364031; RRID: AB_2894493
CD7–PE	BioLegend	Cat# 343106; RRID: AB_1732011
HDAC2	ThermoFisher	Cat# PA1-861; RRID: AB_2118520

**Chemicals, peptides, and recombinant proteins**		

CHIR99021	STEMCELL Technologies	Cat# 72052
all-trans retinal	Sigma-Aldrich	R2500-25mg
Cas9-NLS protein	University of California QB3 Macrolab	N/A

**Critical commercial assays**		

ALDEFLUOR™ Kit	STEMCELL Technologies	Cat# 01700
StemSpan™ T cell Generation Kit	STEMCELL Technologies	Cat# 09940
Chromium Next GEM Single Cell Multiome ATAC + Gene Expression Reagent Bundle	10x Genomics	PN-1000283

**Deposited data**		

Single cell multiome raw and processed data	This paper	NCBI SRA: PRJNA1272807

**Experimental models: Cell lines**		

Human embryonic stem cell line H1	WiCell Research Institute	WiCell Cat# wa01; RRID: CVCL_9771
Human tonsil B cell-derived iPSC line	This paper	N/A
Lenti-X 293T cells	Takara Bio USA	Cat# 632180

**Oligonucleotides**		

Sequences of guide RNAs and PCR primers to generate amplicons for Nanopore sequencing used in Cas9-knockout validation experiments	This paper	Table S1

**Recombinant DNA**		

lentiGuide-mCherry	Addgene	https://www.addgene.org/217005/
psPax2	Addgene	https://www.addgene.org/12260/
VSV.G	Addgene	https://www.addgene.org/12259/

**Software and algorithms**		

GraphPad Prism 10	GraphPad	https://www.graphpad.com/
Biorender	Biorender	https://www.biorender.com/
FlowJo™ v10	Waters Corporation	https://www.flowjo.com/

### Experimental model and study participant details

#### Ethics statement

Tonsil samples were obtained from a female individual undergoing elective tonsillectomy (Banner-University Medical Center). The procedure was medically indicated and not a research intervention. Samples had no identifying information besides sex and would otherwise have been discarded. The Human Research Protections Office of the University of Arizona and the Non-Research Data Use Committee of Banner Health considered the work to be non-human subjects research. Research involving human pluripotent stem cells conform to Category 1A as defined by the ISSCR Guidelines for Stem Cell Research and Clinical Translation (2021).

#### iPSC line

Bilateral tonsils were combined and mechanically dissociated with a mortar and pestle. 4 x 10^6^ CD19^+^ IgM+ were sorted and cultured in RPMI +10% fetal calf serum for three days in the presence of 10 ng/mL of CD40L (Peprotech), 15 ng/mL IL-2 (Peprotech), 10 ng/mL IL-6 (Peprotech), and 20 ng/mL IL-10 (Peprotech). Three hundred thousand CD19^+^IgM^+^ cells were infected with Sendai virus encoding OCT4, SOX2, KLF4, and MYC (CytoTune-iPS 2.0 Reprogramming Kit, Thermo Fisher Scientific) and cultured for 3 days in a xeno-free, blood-based medium on Matrigel (Churko et al., 2013). Cells were subsequently transferred to E7 medium for 12 days, followed by E8 medium until colony formation. Individual colonies were picked, expanded, and cryopreserved at passage 3. Karyotyping, copy number variant analysis, and mycoplasma testing was performed on these stocks. For human embryonic stem cell (hESC) experiments, H1 cells were used (WA01, NIH registry 10–0043, WiCell).

### Method details

#### Cell culture and differentiation of hPSCs

H1 hESCs and induced pluripotent stem cells (iPSCs) were maintained in feeder-free conditions on Matrigel-coated plates in mTeSR Plus medium at 37°C with 5% CO2. Cells were passaged every three to four days at a 1:6 ratio using ACCUTASE. Stocks of cells were frozen in mFRESR according to manufacturer’s instructions (Stem Cell Technologies). H1 stocks were passage 51 and iPSC stocks were passage 3. All experiments were performed within 10 passages. For thawing, cryopreserved cells were warmed in a 37°C water bath and then transferred to prewarmed DMEM, spun, and resuspended and plated in pre-warmed mTESR containing 10μM ROCK inhibitor Y-27632 (Cayman Chemicals). After 24 h, the media was changed to mTESR without ROCK inhibitor. For differentiation, hPSCs were scraped off plates using Gentle Cell Dissociation Reagent and plated onto ultra-low attachment plates to form embryoid bodies (EBs). On day 0, differentiation medium made up of 73.5% IMDM, 25% Ham’s F12, 1% N2, 0.5% B27, and 0.05% BSA (Thermo Fisher) is supplemented with GlutaMAX (2 mM, Thermo Fisher), monothioglycerol (MTG; 0.45 mM, Sigma-Aldrich), ascorbic acid (50 ng/mL), transferrin (1.5 mg/mL, Sigma-Aldrich), bone morphogenetic protein 4 (BMP4; 10 ng/mL), basic fibroblast growth factor (bFGF; 5 ng/mL), and CHIR99021 (3 μM) as indicated. On day 2, SB-431542 (6 μM) was added. On Day 3, EBs were changed to StemPro-34 medium supplemented with GlutaMAX, MTG, ascorbic acid, transferrin, and bFGF, as above, with additional vascular endothelial growth factor (VEGF; 15 ng/mL) and all-trans retinal (RAL; 500 nM, Sigma-Aldrich). On day 6, interleukin 6 (IL-6; 10 ng/mL), insulin-like growth factor 1 (IGF-1; 25 ng/mL), interleukin 11 (IL-11; 5 ng/mL), stem cell factor (SCF; 100 ng/mL) and erythropoietin (EPO; 2 U/mL) were added. On day 10 and 13, FLT3L (5 ng/mL) and interleukin 7 (IL-7; 5 ng/mL) were added. All differentiation cultures were maintained at 37°C with 5% CO2. Sterility from bacteria and fungi were assessed visually. Unless indicated otherwise, all reagents and human recombinant factors were obtained from STEMCELL Technologies. Pluripotency was confirmed using the STEMdiff Trlineage Differentiation kit (Stem Cell technologies) followed by CXCR4, BRACHYURY, SOX17, NESTIN, and PAX6 to identify all three germ layers (Pizzato et al., 2024).

#### Flow cytometry analysis

On day 3, EBs were dissociated into single cells using 0.25% Trypsin-EDTA for ten minutes. Cells were first stained for aldehyde dehydrogenase expression using the ALDEFLUOR Kit (StemCell Technologies, Cat# 01700) according to the manufacturer’s protocol. Control samples were established using diethylaminobenzaldehyde (DEAB), an ALDH inhibitor. Cells were then washed and stained in ALDEFLUOR Assay buffer with KDR–PE-Cy7 (clone 7D4-6, BioLegend, Cat# 359912, 1:100) for 45 min, and dead cells were excluded using DAPI staining. Cells were analyzed on an LSRFortessa (BD) cytometer. Fluorescence-activated cell sorting (FACS) was performed on a 5-laser BD FACS ARIA II.

#### T cell differentiation

Day 16 EBs were harvested and dissociated into single cells for fluorescence-activated cell sorting (FACS) using 0.25% Trypsin-EDTA for ten minutes, followed by Collagenase Type II for 30 min. A total of 500–2000 FACS-sorted CD34^+^ cells were added to individual wells of a 24-well plate coated with StemSpan Lymphoid Differentiation Coating Material (StemCell Technologies). Cells were cultured according to the manufacturer’s protocol for 14 days in StemSpan SFEM II medium supplemented with StemSpan Lymphoid Progenitor Expansion Supplement (StemCell Technologies). Following 14 days of differentiation, cells were analyzed using a BD-Fortessa flow cytometer (BD Biosciences). The antibodies used include CD13–PE-Dazzle 594 (clone WM15, BioLegend, Cat# 301720; 1:100), CD14–PE-Cy7 (clone M5E2, BioLegend, Cat# 301814; 1:100), CD45–FITC (clone 2D1, BioLegend, Cat# 368508; 1:100), CD5–PE-Cy5 (clone L17F12, BioLegend, Cat# 364031; 1:100), and CD7–PE (clone CD7-6B7, BioLegend, Cat# 343106; 1:100). Cells were sorted with a FACSAria II (BD) cell sorter and analyzed on an LSRFortessa (BD) cytometer.

#### Combined single-cell ATAC-seq and RNA-seq

Single-cell suspensions were generated from Day 0 undifferentiated H1 hESCs, and Day 1.5 and Day 3 EBs, both with and without 3 μM CHIR99021 treatment. 10,000 nuclei per sample were processed and isolated using the Nuclei EZ Prep kit (Sigma-Aldrich) according to the manufacturer’s protocol. Libraries for both ATAC-seq and RNA-seq were prepared using the Chromium Next GEM Single Cell Multiome ATAC + Gene Expression Reagent Bundle (10x Genomics, PN-1000283). Both modalities were obtained from the same nuclei. Gene expression measurements reflect nuclear RNA captured during the multiome assay. Libraries were prepared with i7 indices, multiplexed, and sequenced in partial lanes of the Illumina NovaSeq X Plus Series (PE150) to obtain 25,000 paired-end reads per nuclei. Unique sequences in each i7 index were then used for demultiplexing. Raw sequencing data from both ATAC-seq and RNA-seq were processed using Cell Ranger (10x Genomics) for alignment to the hg38 reference genome, filtering, and generating expression and accessibility matrices. These matrix files were then imported into RStudio for data processing which includes filtering out low quality cells, normalizing gene expression data using SCTransform and DNA accessibility data using latent sematic indexing (LSI). We then integrated all datasets into a single merged Seurat object following a previously described pipeline (https://stuartlab.org/signac/articles/pbmc_multiomic). We performed linear dimensional reduction on the integrated dataset to create Uniform Manifold Approximation and Projection (UMAP) graphs to visualize the clustering of cells based on both chromatin accessibility and gene expression profiles. Pseudotime analysis was then conducted using Monocle 3.0. Cells were ordered based on their differentiation status from undifferentiated pluripotent states (day 0) through intermediate (day 1.5) to differentiated progenitors (day 3). Cell trajectories were visualized on the UMAP embedding. Single-cell libraries were generated from one independent differentiation experiment. Key findings from the multiome analysis were independently validated through functional perturbation experiments, including CRISPR-mediated knockout and lentiviral overexpression as described in this study.

#### Gene regulatory network (GRN) and perturbation modeling using CellOracle

CellOracle (v2.3.2) was used to infer transcription factor-driven regulatory networks. First, CellOracle identifies accessible regulatory elements (enhancers/promoters) from scATAC-seq data. Next, these regions are scanned for known transcription factor (TF) binding motifs, which define all possible TF-target gene interactions. CellOracle then integrates scRNA-seq data to retain only TF-target interactions supported by observed expression patterns. This results in dataset-specific gene regulatory networks (GRNs) that were used to identify TFs essential for development of a particular cell cluster. In silico perturbation simulations, overexpression (OE) and knockout (KO), were then used to predict regulatory impacts of a TF on differentiation trajectories. Perturbation Scores (PS) were calculated for each factor, quantifying their predicted ability to promote or inhibit differentiation.

#### CRISPR/Cas9 gene editing of human embryonic stem cells

H1 hESCs were cultured under standard feeder-free conditions described above. 24 h before electroporation, cells were moved to antibiotic-free mTeSR Plus medium containing CloneR2 (Stem Cell Technologies). Cells were then harvested and electroporated using the Neon Transfection System (Thermo Fisher) with ribonucleoprotein (RNP) complexes consisting of recombinant Cas9 protein (40 μM, QB3 MacroLab) and synthetic single-guide RNAs (sgRNAs; 160 μM, IDT) targeting HDAC2, ID3, TCF4, TCF12 individually, or TCF4 and TCF12 simultaneously (double KO). B2M-targeting sgRNAs served as a negative control (Table S1). Electroporated cells were plated onto Matrigel-coated plates containing antibiotic-free mTeSR Plus medium supplemented with CloneR2 for 24 h, then maintained as described above without CloneR2. Edited cells were then subjected to EB differentiation in the presence of CHIR99021 to generate mesodermal progenitors.

#### Genomic DNA extraction, PCR amplification, sequencing, and CRISPR editing efficiency analysis

Genomic DNA from FACS-isolated populations (Day 3 KDR+AF+ and KDR+AF- cells) and Day 0 undifferentiated cells was extracted using the Genomic DNA Blood/Cultured Cell Kit (IBI Scientific IB47207). Gene-targeted regions were PCR-amplified using primers specifically designed around sgRNA target sites, producing amplicons between 200 and 1,000 bp (Table S1). PCR products were purified using the Gel/PCR DNA Fragments Extraction Kit (IBI Scientific IB47082) and sent to Plasmidsaurus for amplicon sequencing using the Oxford Nanopore platform. Quantification of frameshift mutation frequency at each CRISPR target site was performed using CRISPResso2 (Clement et al., 2019). Amplicon sequencing reads were aligned to the reference sequence and indel frequencies were calculated within a ±5 bp window surrounding the predicted Cas9 cut site. Frameshift frequency was defined as the proportion of reads containing insertions or deletions predicted to disrupt the coding frame relative to total aligned reads. Frameshift mutation frequency was compared between sorted Day 3 KDR+ AF+ and KDR+ AF- populations.

#### Small-molecule inhibitor screen

H1 hESCs were seeded into 96-well ultra-low attachment plates to induce embryoid body (EB) formation in Day 0 differentiation medium as described above. Small-molecule inhibitors (Cayman Chemical Item #11076, Batch #0605203, comprehensive list in Table S2) targeting histone acetyltransferases, deacetylases, methyltransferases, and demethylases were added to the cultures at 10 μM final concentration using the Biomek FX liquid handling system. DMSO was used as a vehicle control. On Day 3 of differentiation, EBs were dissociated into single cells and stained for ALDEFLOUR, as described above. Flow cytometric analysis was performed using the 96-well autosampler of the Attune cytometer.

#### Lentivirus construct design, production, and transduction

HDAC2, ID1, and ID3 genes were each cloned without stop codons into constitutive lentiviral vectors downstream of the E1Fα promoter and in-frame with a downstream T2A-mCherry cassette. Lenti-X 293T cells (Takara Bio USA) were cultured at 37^o^C with 5% CO_2_ in DMEM with 10% FBS, nonessential amino acids, Glutamax, sodium pyruvate, and penicillin/streptomycin. Cells were transfected at approximately 80% confluency in 10 cm^2^ tissue culture plates using 30 μL GeneJuice Transfection Reagent (Sigma-Aldrich) with 5 μg of lentiviral vector, 3.25 μg psPax2 (Addgene 12260), and 1.75 μg VSV.G (Addgene 12259). Medium was changed 6 to 8 h post-transfection, and viral supernatant was harvested 48 and 72 h later. 12 mL of viral supernatant was mixed with 3mL of 25% polyethylene glycol 8000 (Sigma-Aldrich) in PBS and incubated overnight at 4^o^C. This mixture was centrifuged at 3,000 x g for 20 min. Supernatant was discarded and the pellet was resuspended in 100μL PBS. Aliquoted lentivirus was stored at −80^o^C until time of use. 5 to 10 μL was used to transduce one well of H1 hESCs in a six-well plate.

Some figure panels were created with BioRender.com.

### Quantification and statistical analysis

Statistical significance was evaluated using a *t* test (alpha = 0.05) in Figures 1C and 1D. In all other figures, one-way analysis of variance (ANOVA, alpha = 0.05) was performed to test for statistical significance. Data were presented as mean ± standard error of the mean (SEM). Statistical significance was defined as ^∗^*p* < 0.05, ^∗∗^*p* < 0.01, ^∗∗∗^*p* < 0.001, and ^∗∗∗∗^*p* < 0.0001.

## Supplemental information

Document S1. Figures S1–S7 and Table S1

Table S2. Summary of drug screen results on AF+ mesoderm using small molecule epigenetic modifiers

Document S2. Article plus supplemental information
