# Supplementary material for: WNT signaling in human pluripotent stem cells promotes HDAC2-dependent epigenetic programs and development of retinoic acid-responsive mesoderm
Source: Stem Cell Reports. 2026 Jun 18;21(7):102964. doi: 10.1016/j.stemcr.2026.102964 (PMC13385423; doi:10.1016/j.stemcr.2026.102964)
Supplement: Document S1. Figures S1–S7 and Table S1 [file mmc1.pdf]

Stem Cell Reports, Volume *21*

Supplemental Information

WNT signaling in human pluripotent stem cells promotes HDAC2-dependent epigenetic programs and development of retinoic acid-responsive mesoderm

Bao Q. Thai, Elizabeth J. Sargent, Stephanie A. Luff, Colin A. Fields, Jared M. Churko, Jonathan N. Young, Anthony Bosco, Christopher M. Sturgeon, and Deepta Bhattacharya

Fig. S1

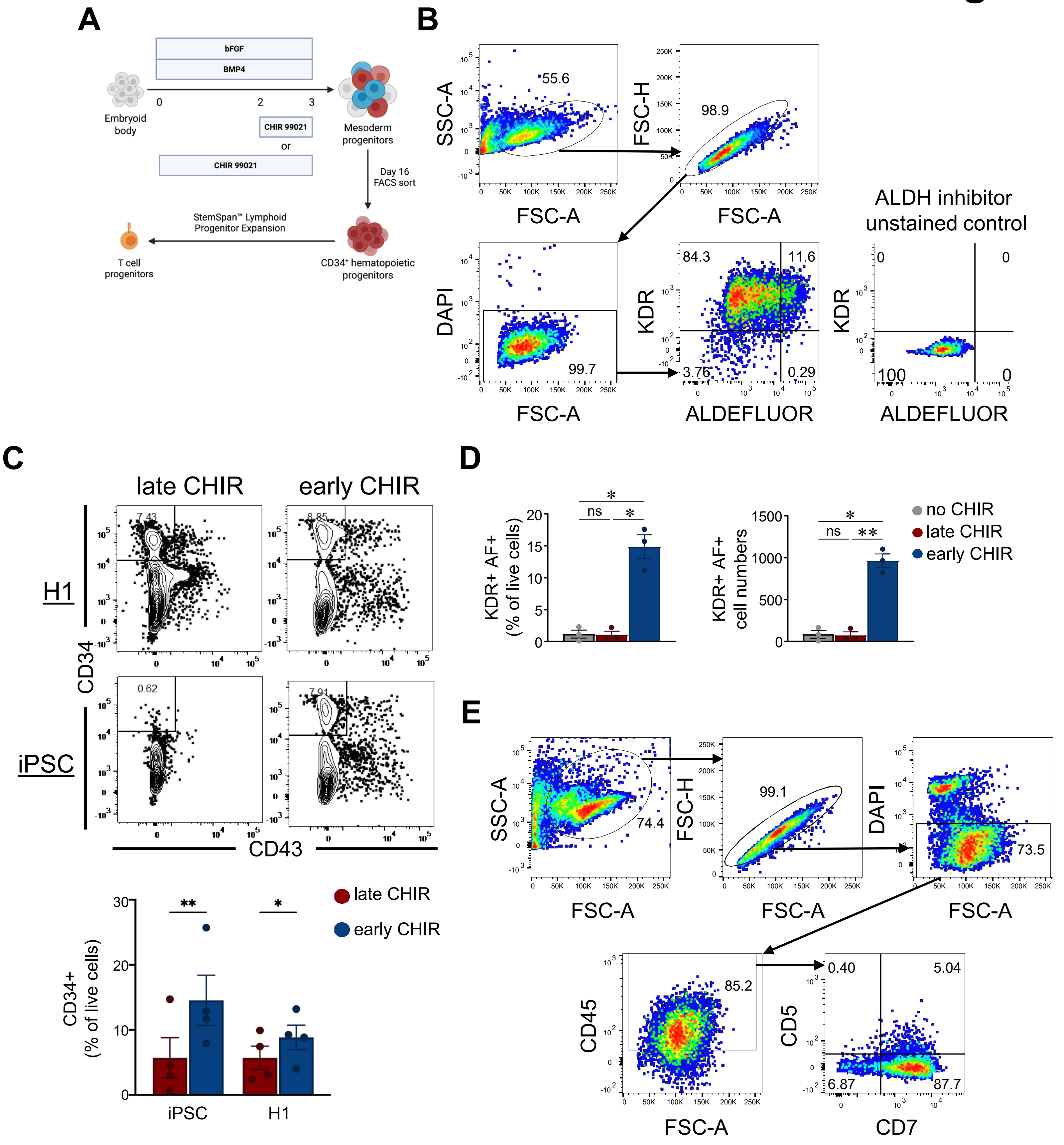
A
bFGF
BMP4
Embryoid body
0
2
3
CHIR 99021
or
CHIR 99021
Mesoderm progenitors
Day 16 FACS sort
StemSpan™ Lymphoid Progenitor Expansion
T cell progenitors
CD34⁺ hematopoietic progenitors
B
SSC-A
FSC-A
55.6
FSC-H
98.9
DAPI
99.7
KDR
ALDEFLUOR
84.3
11.6
3.76
0.29
ALDH inhibitor unstained control
0
0
100
0
C
late CHIR
early CHIR
H1
iPSC
CD34
CD43
7.43
8.85
0.62
7.91
CD34+ (% of live cells)
late CHIR
early CHIR
iPSC
H1
D
KDR+ AF+ (% of live cells)
KDR+ AF+ cell numbers
ns
no CHIR
late CHIR
early CHIR
E
74.4
99.1
73.5
CD45
85.2
CD5
CD7
0.40
5.04
6.87
87.7

Figure S1. Differentiation of mesodermal progenitors from hPSCs and the role of CHIR 99021 in WNT signaling activation. (A) Schematic overview of the three-day differentiation to generate mesodermal progenitors from hPSCs via embryoid body (EB) formation. CHIR 99021 was added on day 0 or day 2 in early CHIR or late CHIR condition, respectively. Day 16 CD34+ progenitors were sorted and subsequently used for T cell assays to assess for definitive hematopoiesis specification. (B) Gating strategy for flow cytometry analysis of KDR and ALDEFLUOR expression in H1- and iPSC-derived progenitors under early or late CHIR conditions. (C) Representative flow cytometry plots showing expression of CD34 and CD43 in H1- and iPSC- derived hematopoietic progenitors on day 16 of EB differentiation, with early or late CHIR treatments, and quantification of the percentage of CD34+ cells in H1- and iPSC-derived cultures. Statistical analysis was performed using 2-way ANOVA with Šídák multiple comparisons test and $*p < 0.05$, $**p < 0.01$, n=4. Data are presented as mean ± SEM. (D) Frequencies and numbers of H1-derived KDR+ ALDEFLUOR+ cells in early CHIR, late CHIR, and no CHIR conditions. (E) Gating strategy for flow cytometry analysis of CD5 and CD7 expression in H1- and iPSC-derived T cell progenitors.

Fig. S2

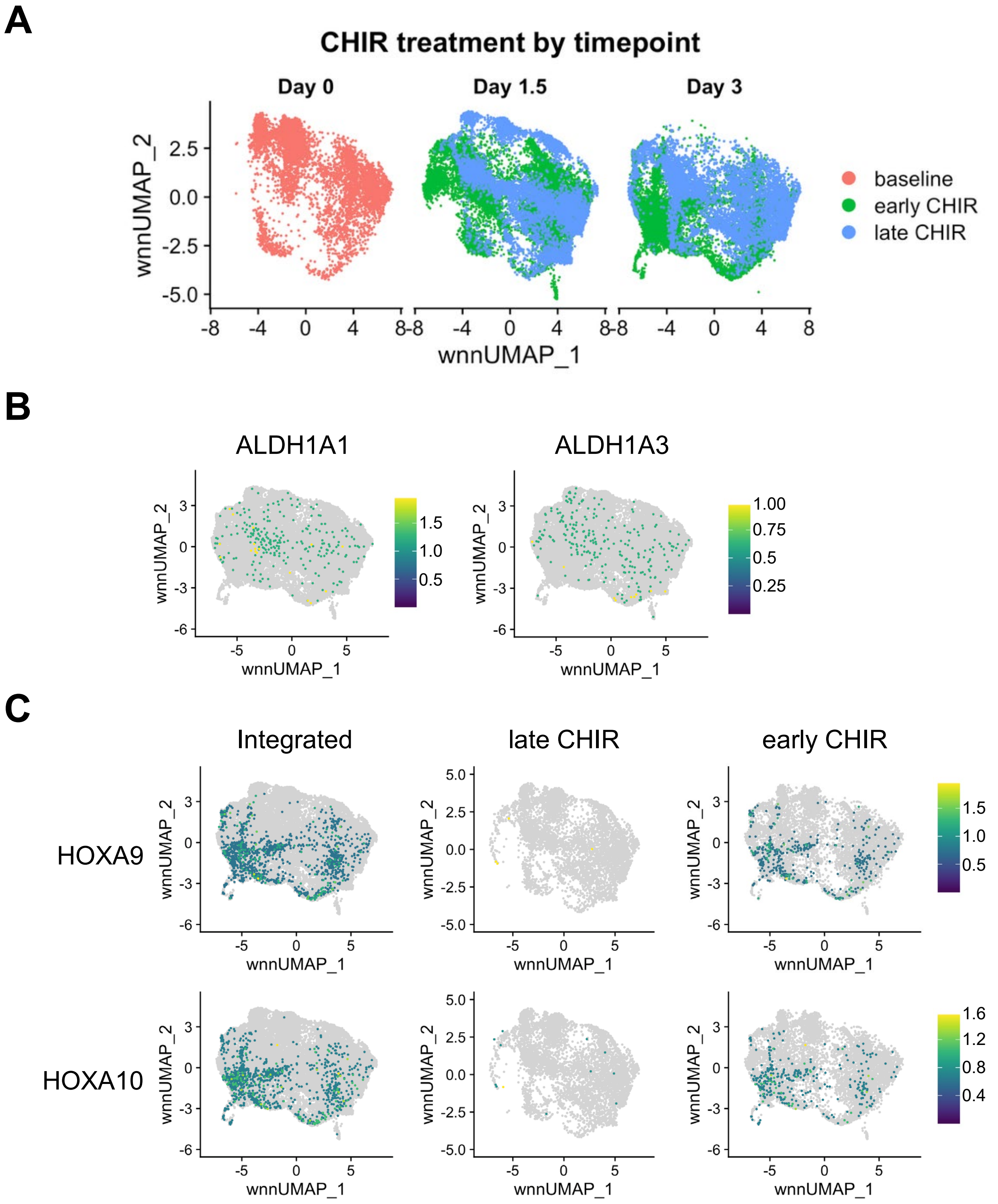
A
CHIR treatment by timepoint
Day 0
Day 1.5
Day 3
wnnUMAP_2
wnnUMAP_1
baseline
early CHIR
late CHIR
B
ALDH1A1
ALDH1A3
C
Integrated
late CHIR
early CHIR
HOXA9
HOXA10

Figure S2. WNT signaling induces transcriptional changes associated with ALDH1A2- and HOXA-specific hematopoietic programs. (A) UMAP plots showing late and early CHIR cells on day 0, day 1.5 and day 3. (B) Relative expression of ALDH1A1 and ALDH1A3*.* (C) UMAP plots showing the relative expression of *HOXA9* and *HOXA10*.

Fig. S3

A

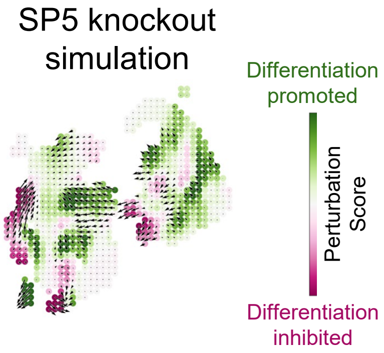

SP5 knockout simulation
Differentiation promoted
Perturbation Score
Differentiation inhibited

B

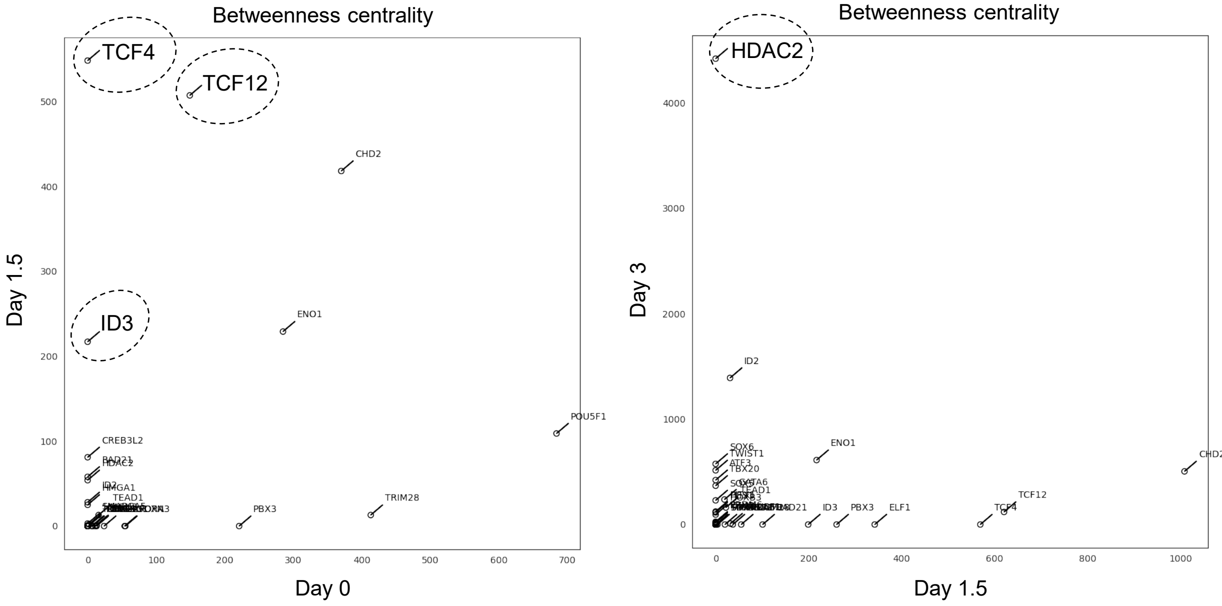

Betweenness centrality
TCF4
TCF12
CHD2
ID3
ENO1
POU5F1
CREB3L2
HDAC2
HMGA1
TEAD1
PBX3
TRIM28
Day 1.5
Day 0
Betweenness centrality
HDAC2
ID2
SOX6
TWIST1
ATF3
TBX20
ENO1
CHD2
TCF12
ID3
PBX3
ELF1
Day 3
Day 1.5

Figure S3. Identification of key regulators in RA-responsive mesodermal differentiation using CellOracle centrality analysis and in silico perturbation simulation. (A) CellOracle perturbation simulation showing the predicted effect of SP5 knockout on differentiation trajectories. Perturbation scores (PS) indicate where SP5 KO promotes (green, positive PS) or inhibits (magenta, negative PS) differentiation. (B) CellOracle network centrality analysis comparing TFs by betweenness centrality at Day 0 vs Day 1.5 cells (left) and Day 1.5 vs. Day 3 cells (right) of early CHIR cultures.

Fig. S4

A

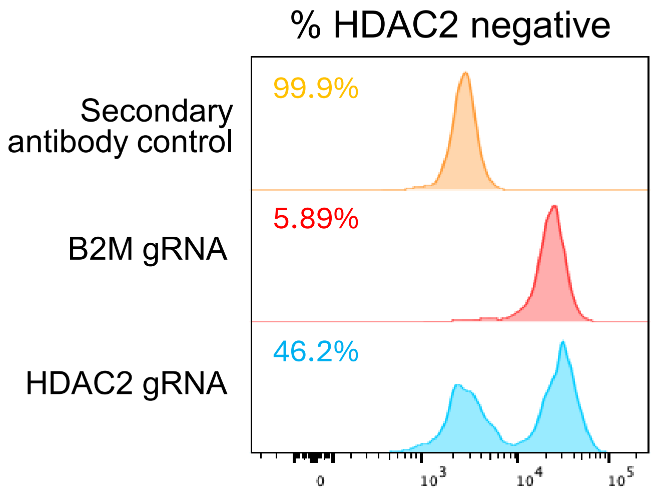
% HDAC2 negative
Secondary antibody control
99.9%
B2M gRNA
5.89%
HDAC2 gRNA
46.2%
0
10^3
10^4
10^5

B

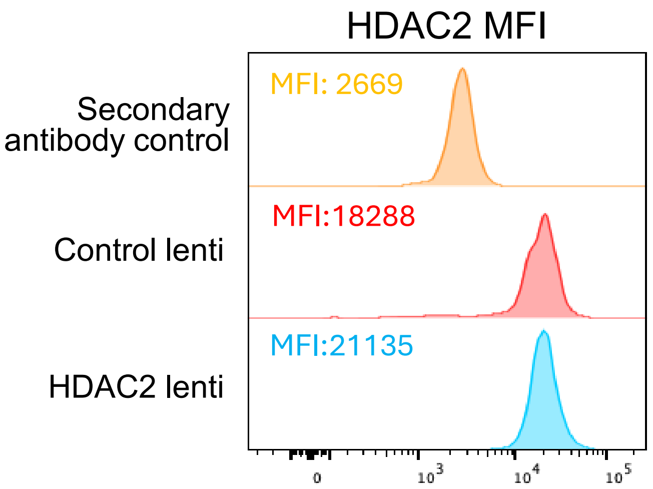
HDAC2 MFI
Secondary antibody control
MFI: 2669
Control lenti
MFI:18288
HDAC2 lenti
MFI:21135
0
10^3
10^4
10^5

Figure S4. Validation of HDAC2 perturbation efficiency at the protein level. (A) Representative flow cytometry histograms showing HDAC2 staining following CRISPR/Cas9-mediated targeting with HDAC2 gRNA versus B2M gRNA as a control. (B) Representative flow cytometry histograms of HDAC2 mean fluorescence intensity (MFI) following lentiviral transduction of HDAC2 lenti versus empty vector lenti as a control.

Fig. S5

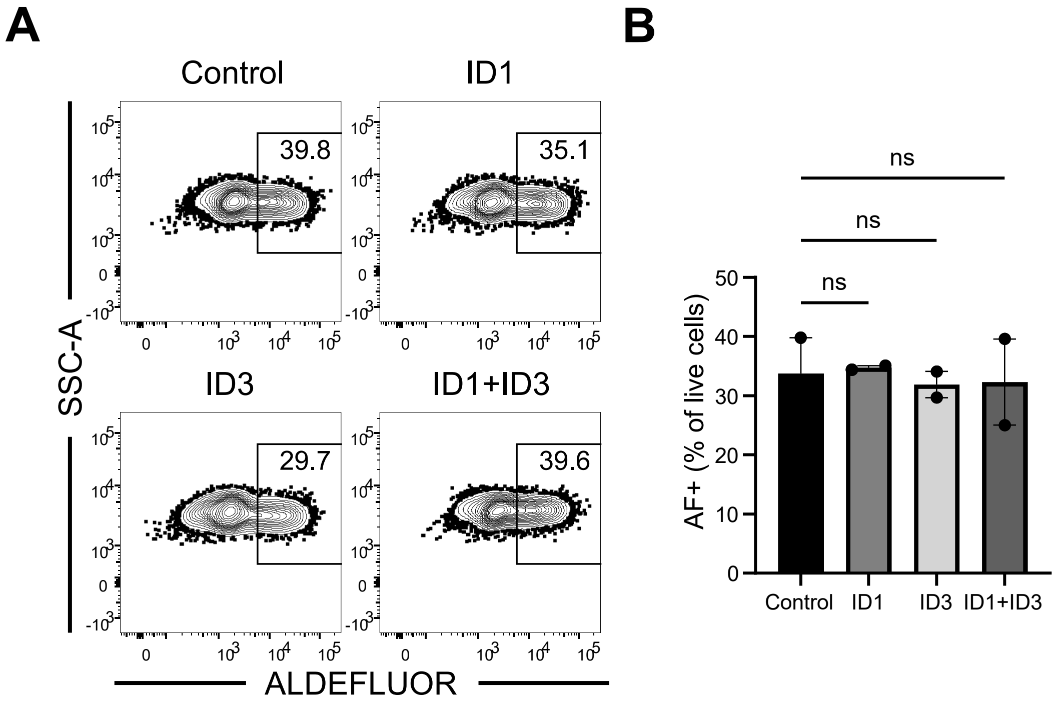
A
Control
ID1
ID3
ID1+ID3
39.8
35.1
29.7
39.6
SSC-A
ALDEFLUOR
B
ns
ns
ns
AF+ (% of live cells)
50
40
30
20
10
0
Control
ID1
ID3
ID1+ID3

Figure S5 relate to Figure 5. Overexpression of ID proteins does not impair generation of AF+ cells. (A) Representative flow cytometry plots showing the differentiation of ALDEFLUOR+ progenitors on day 3 of differentiation. H1 cells were transduced with mCherry-expressing lentiviral vectors encoding ID1, ID3, and ID1 and ID3 in combination, or control (empty vector). Transduced cells were then subjected to the same differentiation protocol as above with CHIR 99021. (B) Quantification of ALDEFLUOR+ progenitor percentage. Statistical analyses were performed using paired 1-way ANOVA with Dunnett multiple comparisons test. Data are presented as mean ± SEM.

Fig. S6

A

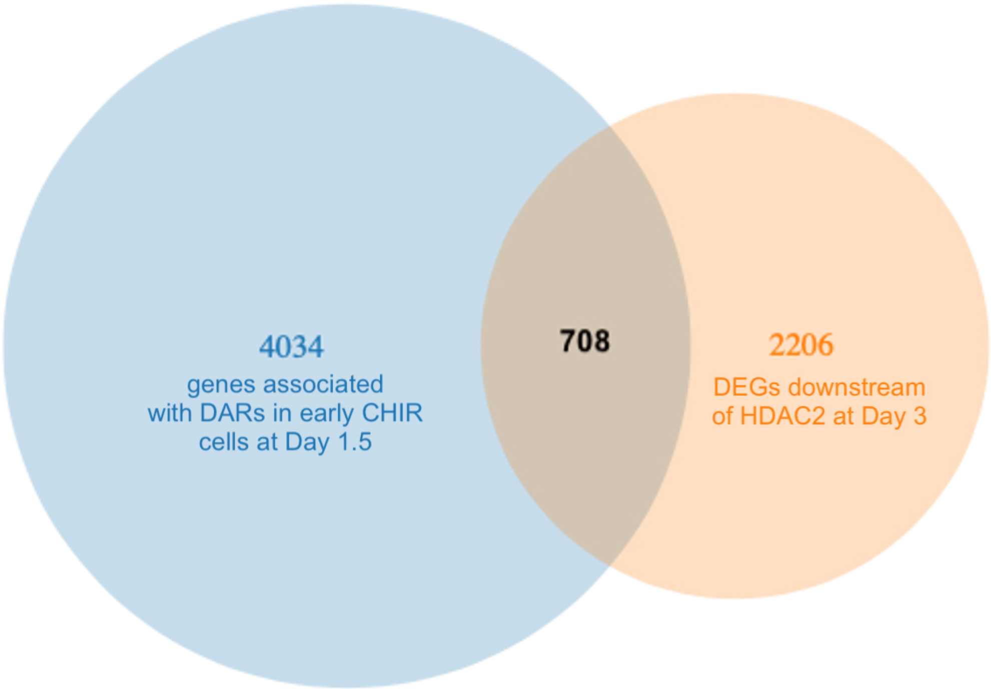
4034
genes associated with DARs in early CHIR cells at Day 1.5
708
2206
DEGs downstream of HDAC2 at Day 3

B

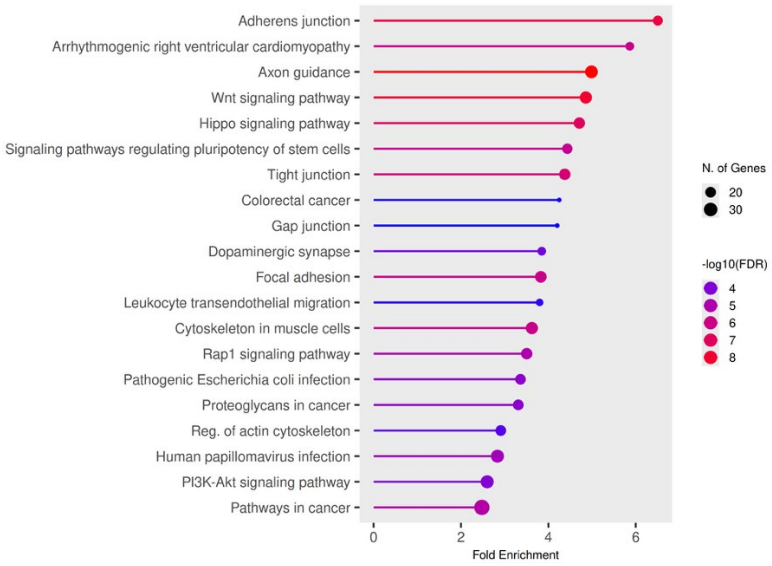
Adherens junction
Arrhythmogenic right ventricular cardiomyopathy
Axon guidance
Wnt signaling pathway
Hippo signaling pathway
Signaling pathways regulating pluripotency of stem cells
Tight junction
Colorectal cancer
Gap junction
Dopaminergic synapse
Focal adhesion
Leukocyte transendothelial migration
Cytoskeleton in muscle cells
Rap1 signaling pathway
Pathogenic Escherichia coli infection
Proteoglycans in cancer
Reg. of actin cytoskeleton
Human papillomavirus infection
PI3K-Akt signaling pathway
Pathways in cancer
0
2
4
6
Fold Enrichment
N. of Genes
20
30
-log10(FDR)
4
5
6
7
8

Figure S6. Integration of early CHIR-driven chromatin accessibility with HDAC2-dependent transcriptional programs. (A) Venn diagram showing the overlap between genes associated with differentially accessible regions (DARs) in early CHIR99021-treated cells at Day 1.5 (4,034 genes) and differentially expressed genes (DEGs) predicted to lie downstream of HDAC2 at Day 3 (2,206 genes). A total of 708 genes are shared between these datasets. (B) Pathway enrichment analysis of the overlapping gene set reveals enrichment for WNT signaling and pathways related to cell adhesion and junction organization. Dot size represents the number of genes. Color indicates -log10(FDR).

Fig. S7

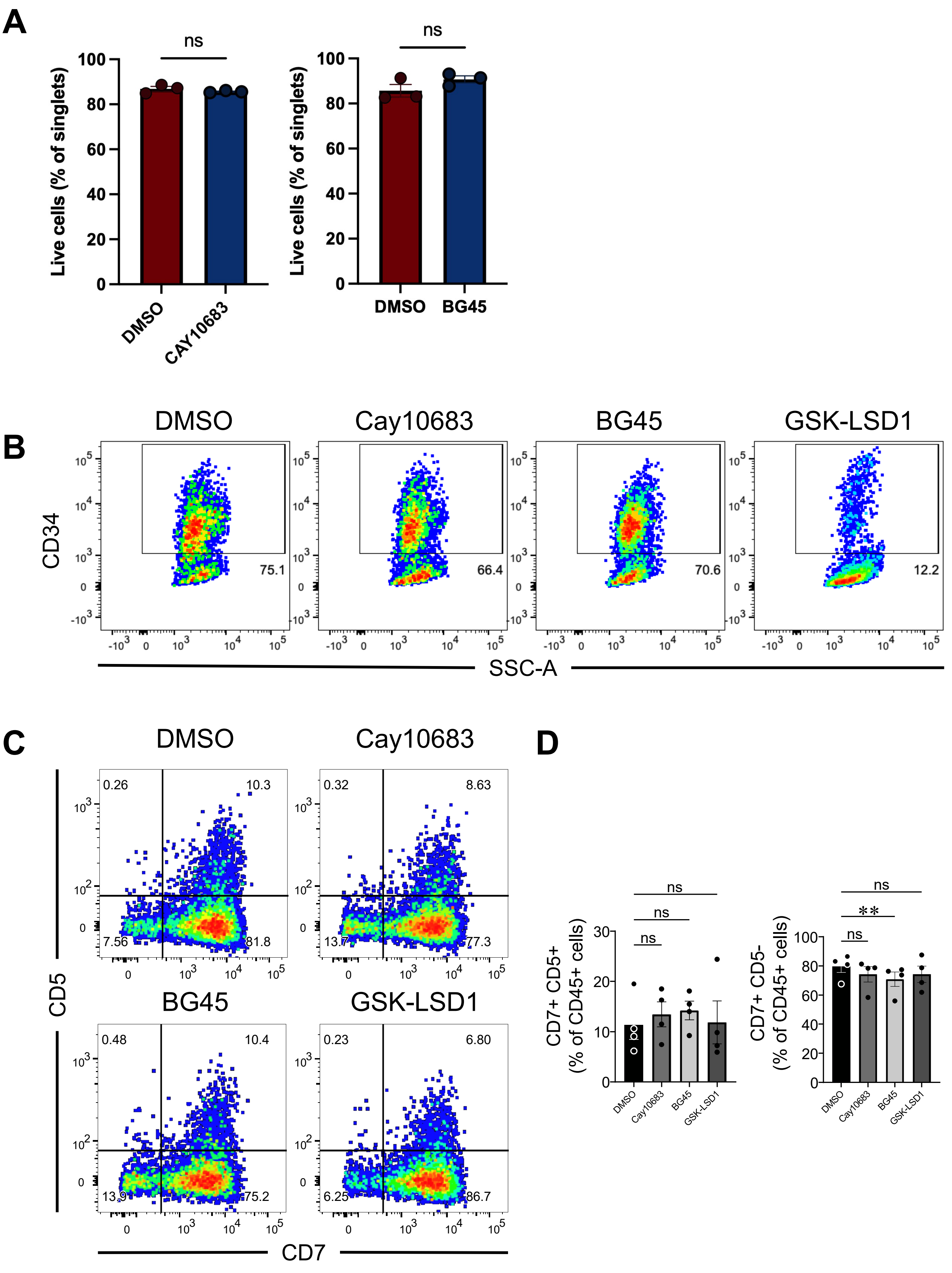
A
ns
Live cells (% of singlets)
DMSO
CAY10683
BG45
B
DMSO
Cay10683
BG45
GSK-LSD1
CD34
75.1
66.4
70.6
12.2
SSC-A
C
CD5
CD7
0.26
10.3
7.56
81.8
0.32
8.63
13.7
77.3
0.48
10.4
13.9
75.2
0.23
6.80
6.25
86.7
D
CD7+ CD5+ (% of CD45+ cells)
CD7+ CD5- (% of CD45+ cells)
ns
**
Cay10683

Figure S7. Effects of small molecule modulators on cell viability, CD34, and T cell differentiation. (A) Quantification of live cells (% of singlets) following treatment with DMSO, Cay10683 or BG45. Statistical analysis was performed using 2-tailed t-test. Data are presented as mean ± SEM, n=3. (B) Representative flow cytometry plots showing CD34 expression following treatment with DMSO, Cay10683, BG45, or GSK-LSD1. (C) Representative flow cytometry plots showing CD7 and CD5 expression following treatment with CAY10683, BG45, or GSK-LSD1 compared to DMSO control. (D) Quantification of CD7+ CD5+ and CD7+ CD5- populations (as a percentage of CD45+ cells) is shown. Statistical analyses were performed using one-way ANOVA with multiple comparisons, **$p < 0.01$, n=4. Data are presented as mean ± SEM, with each point representing an independent differentiation.

Table S1. Sequences of guide RNAs and PCR primers to generate amplicons for Nanopore sequencing used in Cas9-knockout validation experiments.

Name	Sequence	Source
B2M gRNA	GGCCACGGAGCGAGACAUCU	IDT
HDAC2 gRNA	GAUGUAUCAACCUAGUGCUG	IDT
TCF4 gRNA	CGAUGGAAAGUGGACAUCGG	IDT
TCF12 gRNA	AGUCGAUUAGGAGCCCAUGA	IDT
ID1 gRNA	CGGCAAGACAGCGAGCGGUG	IDT
ID3 gRNA	AUGUCGUCCAGCAAGCUCAG	IDT
B2M fwd seq	CGCTGGCTTGGAGACAGG	Sigma-Aldrich
B2M rev seq	GCAGCAGACAGGCTTACCCG	Sigma-Aldrich
HDAC2 fwd seq	GGTGCTGGAAAAGGCAAATACTATGCTG	Sigma-Aldrich
HDAC2 rev seq	CTGAACACATGAAGATACTGAGACACCAG	Sigma-Aldrich
TCF4 fwd seq	CCTCCACAGTTGATGCAAACCC	Sigma-Aldrich
TCF4 rev seq	GTAGCTCACTAGTCACTGATGGCTC	Sigma-Aldrich
TCF12 fwd seq	CCCTAGGGTTTTACAGACAGCCC	Sigma-Aldrich
TCF12 rev seq	CCTGTCCCTCCTGGACAAAG	Sigma-Aldrich
ID1 fwd seq	CGTATCTGCTTCGGGCTTCC	Sigma-Aldrich
ID1 rev seq	GCACGTAATTCCTCTTGCCCC	Sigma-Aldrich
ID3 fwd seq	GGCACCTCTGGACTCACTC	Sigma-Aldrich
ID3 rev seq	CGAGTCAGTGGCAAAAGCTCC	Sigma-Aldrich
